# Real World Analysis of Small Cell Lung Cancer Patients: Prognostic Factors and Treatment Outcomes

**DOI:** 10.3390/curroncol28010036

**Published:** 2021-01-08

**Authors:** Sarah Sharman Moser, Jair Bar, Inna Kan, Keren Ofek, Raanan Cohen, Nikhil Khandelwal, Varda Shalev, Gabriel Chodick, Nava Siegelmann-Danieli

**Affiliations:** 1 Maccabitech, Maccabi Institute for Research & Innovation, Maccabi Healthcare Services, Tel Aviv 6812509, Israel; Shalev_V@mac.org.il (V.S.); hodik_g@mac.org.il (G.C.); danieli_na@mac.org.il (N.S.-D.); 2 Sheba Medical Center, Institute of Oncology, Tel Hashomer, Ramat Gan 5262000, Israel; Yair.Bar@sheba.health.gov.il; 3 Sackler Faculty of Medicine, Tel Aviv University, Tel Aviv 6997801, Israel; 4 Abbvie Biopharmaceuticals Ltd., Hod Hasharon 4524075, Israel; inna.kan@abbvie.com (I.K.); keren.ofek@abbvie.com (K.O.); raanan.cohen@abbvie.com (R.C.); 5 Abbvie Inc., Chicago, IL 60064, USA; nikhil.khandelwal@Abbvie.com

**Keywords:** SCLC, survival, observational study, platinum sensitive, limited stage

## Abstract

In this observational study, we assessed treatment patterns and prognostic factors in patients with small cell lung cancer (SCLC) in a large state-mandated healthcare organization in Israel. *Methods:* All incident cases with histologically confirmed SCLC who initiated systemic anti-cancer treatment between 2011 and 2017 were identified. Treatment patterns and overall survival (OS) were evaluated for each line of therapy. *Results:* A total of 235 patients were identified (61% male, median age 64 years, 95% ever smokers, 64% had extensive stage). The first-line treatment was platinum–etoposide regimen for 98.7% of the cohort. The second and third-line regimen were given to 43% and 12% of patients, respectively. Mean OS for extensive and limited stage patients was 9.1 and 23.5 months respectively. In a multivariable model, increased risk for mortality was observed among patients with an ECOG performance status (PS) of 2 compared to a PS of 0–1 for the extensive stage patients (Hazard ratio (HR) = 1.63, 95% confidence ratios (CI): 1.00–2.65); and for males compared to females for the limited stage patients (HR = 2.17; 95% CI: 1.12–4.20). Regarding all 2nd line patients in a multivariable model incorporating relevant confounding factors, demonstrated a significantly better outcome with platinum–based regimens compared to topotecan. Median survival after initiation of 2nd line in platinum-sensitive patients was longer (*p* = 0.056) for those re-challenged with platinum–based regimen (n = 7): 6.8mo (6.1-not reported (NR)), compared with those switched to a different treatment (n = 27): 4.5 mo (2.6–6.6) for extensive stage patients, and a non-significant difference was also observed for limited stage patients. *Conclusion:* To our knowledge, this is one of the largest real-world studies of SCLC patients. OS for SCLC patients was similar to that reported in clinical trials. PS for extensive stage patients and sex for limited stage patients were significant correlates of prognosis. Re-challenge of the platinum–based doublet was associated with longer OS compared to switching treatment in extensive stage patients.

## 1. Introduction

Small cell lung cancer (SCLC) is an aggressive smoking-associated malignancy with rapid growth and early metastatic dissemination [1] that accounts for 10% to 15% of all diagnoses of lung cancers [2,3,4,5,6]. Almost two-thirds of SCLC patients have extensive stage disease at presentation, with a median survival of 7 to 11 months and only 1% being alive at 5 years [7,8,9].

Patients with limited stage disease are treated with concurrent chemotherapy and chest irradiation, whereas extensive stage patients are traditionally treated with chemotherapy alone. First-line (L1) chemotherapy with cisplatin or carboplatin with etoposide has been the standard of care for several decades [10], with no changes besides the recent incorporation of atezolizumab to platinum–based regimen in patients with extensive stage disease [11,12,13,14]. Prophylactic brain irradiation mostly for responding patients with limited disease was part of the standard of care [15,16] but has recently been challenged [17]. Consolidate radiotherapy for highly responding extensive disease is another controversial topic [18,19]. Despite high sensitivity to initial chemotherapy and radiotherapy, most patients with either limited or extensive tumors relapse relatively early [20,21] and cure rates remain low [22].

Recurrent disease has limited treatment options. Second-line (L2) systemic chemotherapy is the standard of care [23,24,25,26] for medically fit patients, with regimen choice partly determined by prior response to L1 platinum-based regimen [26,27,28]. Sensitive disease is defined in this tumor type by initial response to L1 platinum-based treatment and a post-treatment progression-free interval of at least 90 days [23]. Patients with sensitive tumors have a much greater likelihood of responding to any further systemic treatment than resistant patients [29,30,31] and are often candidates for re-challenge with platinum-based regimens; all relapsed patients are candidates for topotecan therapy [8,32,33,34]. Amrubicin is another L2 treatment option approved and utilized in Japan [20]. A small fraction of patients may benefit from immunotherapy treatment with the anti-PD-1 agent nivolumab, mostly in L3, as treatment benefit was not seen in a randomized trial of L2 patients [11,35,36,37]. Chemotherapy for L3 may be considered for a limited group of medically fit patients, and studies suggest survival of 3.8–4.7 months [38,39] Thus, treatment options for relapsing cases are limited, confer mostly short-term clinical benefit, and are mostly based on data from single-institution retrospective reviews or small, single-arm trials [40,41,42,43].

Considering the developments in the field of immune-oncology, updated, multi-institutional real-world data have a high value for guiding design of future studies and for critical evaluation of novel therapies. We are aware of only one cohort of real-world patients with extensive-stage disease treated with chemotherapy alone during the years 2009–2013 [44]. We describe here a retrospective cohort study of the characteristics, treatment patterns and overall survival (OS) of SCLC patients with both limited and extensive-stage, including immunotherapy-treated patients in recent years.

## 2. Materials and Methods

### 2.1. Data Source

This retrospective cohort study was conducted using the computerized databases of Maccabi Healthcare Services (MHS), the second largest of four nationwide healthcare insurer-provider organizations. Membership in MHS is free and every citizen is eligible to register as a member for whichever health fund he or she chooses, without limitations of preconditions or age. MHS has approximately 2.1 million members, representing a quarter of the population and shares similar sociodemographic characteristics with the general population [45]. The MHS database contains longitudinal clinical data that are automatically collected since 1993 for a stable population (with less than 1% of members moving out each year), including laboratory results from a single central laboratory, pharmacy prescription and purchase data, hospitalizations, procedures and consultations. MHS uses the International Classification of Diseases, Ninth Revision, Clinical Modification (ICD-9-CM) coding systems, as well as self-developed coding systems to provide more granular diagnostic information. Procedures are coded using Current Procedural Terminology codes. MHS has developed several computerized registries of major chronic diseases, such as cardiovascular disease, oncologic diseases, diabetes mellitus, and osteoporosis, in order to improve the quality of chronic care delivery to its members. Such registries are continuously updated, and they identify patients via automatic search formulas, as opposed to being dependent upon active reporting by physicians [46,47,48].

In addition to the automatic data collection, data that were not available in the main database such as disease staging, imaging results, metastases and eastern cooperative oncology group performance status (PS), were manually extracted by trained data extractors from individual PDF files of patient data in the MHS medical health records.

### 2.2. Study Population

In this retrospective cohort study we identified MHS members aged 18 years or above with a SCLC diagnosis, based on the national cancer registry (which uses diagnosis data from the Israel Ministry of Health, linked to cancer medication approvals and pathology reports from MHS) [49] or with a SCLC diagnosis code in the MHS electronic medical records, and confirmed by manual review of pathology reports. To be included in the study patients had to have received at least one systemic chemotherapy regimen.

Patients were included if they received the first systemic treatment for SCLC between 1 January 2011 and 31 December 2017. Index date was set as the date of initiation of first systemic treatment. Patients with less than one year of healthcare registration in MHS before index date were excluded.

Data was collected up to June 2018 to allow for a minimum follow-up of 6 months.

### 2.3. Study Variables

Demographic and clinical data collected included age at index date, sex, socioeconomic status, district, comorbid conditions, weight and smoking. Socioeconomic status was categorized into quartiles based on the poverty index of the member’s enumeration area, as defined by 2008 Israeli National Census [50]. The poverty index is based on several parameters including household income, educational level, crowding and car ownership.

Co-morbidities at baseline were identified using MHS registries (for diabetes mellitus, hypertension, chronic obstructive pulmonary disease, cardiovascular disease, chronic kidney disease and osteoporosis).

Drug purchases and smoking data were cross-linked between the automatic extraction from the database and additional manually extracted data.

### 2.4. Treatment Patterns

Treatment lines were defined according to the sequence of dispensed medications, with information captured both from pharmacy database (for medications approved by MHS), and from hospital medical records (including information on medications provided by private insurance and clinical studies). Addition of a new drug to a current regimen was considered a new treatment line, and cessation of a medication from a combination regimen (likely due to tolerance issues) was considered the same line.

### 2.5. Statistical Analysis

Baseline descriptive characteristics were compared using t tests for continuous variables and chi-square tests for discrete variables.

OS was assessed using all-cause mortality data from the National Insurance Institute, with Kaplan–Meier and Cox proportional hazards regression methods, and plots of expected survival were generated from L1 and L2 treatment initiation. All analyses were performed for extensive and limited stage patients separately, and subdivided into platinum-resistant and platinum-sensitive patients. Platinum sensitivity was defined as an interval of ≥3 months from the end of L1 treatment to the beginning of L2 treatment. Relapse free interval was defined as the interval between last dose of L1 treatment and initiation of L2 therapy (split by 0–3 months, 3–6 months, 6–9 months and 9–12 months).

All analyses were conducted using IBM SPSS Statistics for Windows, Version 22.0. Armonk, NY: IBM Corp, and a *p*-value < 0.05 was considered statistically significant.

The study was approved by the local ethics review board of Bayit Balev Hospital in Israel.

## 3. Results

A total of 235 patients with histologically confirmed SCLC initiated L1 treatment between the years 2011–2017 (inclusive). Median age at start of treatment was 64 years (interquartile range (IQR) 58, 70), 61% were male, 95% ever smokers, and 60% had 0–1 PS at index date (Table 1).

L1 treatment was platinum (carboplatin/cisplatin)-etoposide regimen for 232 patients (98.7%) of the cohort, and three patients (1.3%) received another platinum-based regimen. Median OS for the study population was 11.8 months (95% confidence interval (CI) 9.9–13.7). A hundred and one (43%) patients continued to Line 2 therapy, and 29 (12%) to Line 3.

### 3.1. Extensive Stage Disease

A total of 150 (64%) patients with extensive stage disease were eligible for this analysis and consisted of 38% females, 94% ever smokers, 57% 0–1 PS and 17% brain metastases at index date (Table 1). Mean OS was 9.1 months (95% CI: 8.4–10.3). Additional factors that correlated with better OS in the univariate analysis included better PS (9.6 vs. 8.9 months, PS 0–1 vs. 2, *p* = 0.09, Table 2). In a multivariable model for all-cause mortality, patients with PS = 2 compared to PS = 0–1 had significant increase in mortality with a HR of 1.63 (95% CI: 1.00–2.65, Table 3).

**Figure 1:**
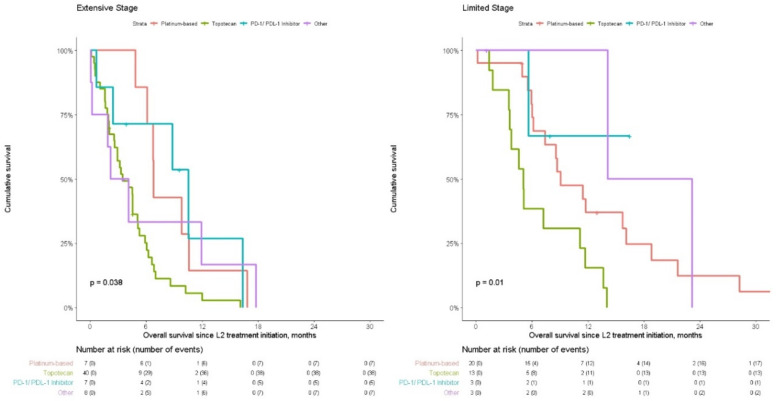
Overall survival from L2 initiation for patients that initiated L2 by treatment, split by extensive (n = 62) and limited (n = 39) stage.

OS from initiation of L2 treatment (n = 62) was 4.54 months (95% CI: 3.19–6.12, Table 2). When split by L2 treatment, OS was 6.81 (6.12-NR), 3.48 (2.63–5.13) and 10.55 (2.43-NR) for platinum-based regimen(n = 7), topotecan (n = 40) and PD-1/PD-L1 inhibitor treatment (n = 7) respectively (*p* = 0.04, Figure 1). Multivariate hazard ratios confirmed a significant difference between platinum-based regimen vs. topotecan L2 treatment (Table 4). Demographic and clinical characteristics were similar between patients that were re-challenged with platinum-based regimen (n = 7) or switched (n = 55) to a different L2 treatment (data not shown).

Extensive stage patients were further stratified by those that were platinum- resistant (*n* = 28) and platinum-sensitive (*n* = 34) to L1 therapy. Demographic and clinical characteristics were similar, however platinum-resistant patients were younger (median age = 61 vs. 65 years, *p* = 0.20), had a higher percentage of males (71% vs. 56%, *p* = 0.32) and were slightly less likely to be smokers (93% vs. 97%, *p* = 0.86) as compared to platinum-sensitive patients (all not significant, data not shown).

Among those that received L2 therapy and were resistant to L1 platinum-basedtherapy (initiated L2 therapy ≤3 months from the cessation of L1 therapy), all patients (n = 28) were switched to a different L2 treatment and did not receive re-challenge with L1 platinum-based regimen: 19 received topotecan (OS = 3.5, 2.1–6.3) and 4 received PD-1/PD-L1 inhibitor treatment (OS = 5.6, 0.66-NR, *p* = 0.43) for the difference between the treatment options (data not shown).

**Figure 2:**
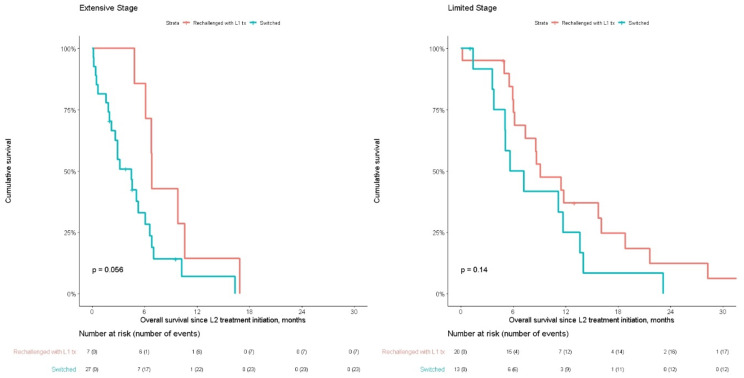
Overall survival from L2 treatment initiation for patients sensitive to L1 therapy (initiated L2 ≥3 months after cessation of L1 therapy), for re-challenge of L1 therapy or switch, split by extensive (n = 34) and limited (n = 33) stage.

Median survival after initiation of 2nd line in platinum-sensitive patients was longer (*p* = 0.056) for those re-challenged with platinum-regimen (n = 7): 6.8mo (6.12-NR), compared with those switched to a different treatment (n = 27): 4.5 mo (2.6–6.6) for extensive stage patients (Figure 2). Of these, 22 received topotecan, 3 received PD-1/PD-L1 inhibitor therapy and 2 received other treatment. Demographic and clinical variables were similar for platinum-sensitive patients between those re-challenged and switched (data not shown).

### 3.2. Limited Stage Disease

A total of 85 (36%) patients with limited stage disease were eligible for this analysis and consisted of 40% females, 98% ever smokers, 66% 0–1 PS and 0% brain metastases at index date (Table 1). Mean OS was 23.5 months (95% CI: 19.4–27.9, Table 2). An additional factor that significantly correlated with better OS in the univariate analysis was female sex (29.6 vs. 21.5 months, *p* = 0.03, Table 2). In a multivariable model for all-cause mortality, males had significant increase in mortality with a HR of 2.17 (95% CI: 1.12–4.20, Table 3).

For patients with relapsed limited stage disease, OS from initiation of L2 treatment (n = 39) was 8.68 months (95% CI: 6.02–14.04, Table 2). When split by L2 treatment, OS was 9.11 (7.4–18.8), 5.06 (3.62-NR) and NR for platinum-based regimen (n = 20), topotecan (n = 13) and PD-1/PD-L1 inhibitor treatment (n = 3) respectively (*p* = 0.01, Figure 1). Multivariate hazard ratios confirmed a non-significant difference between platinum-based regimen vs. topotecan L2 treatment (Table 4). Demographic and clinical characteristics were similar between patients that were re-challenged with platinum-based regimen (n = 20) or switched (n = 19) to a different L2 treatment (data not shown).

Relapsed limited stage patients were further stratified by those that were platinum- resistant (n = 6) and platinum-sensitive (n = 33) to L1 therapy and demographic and clinical characteristics were similar between the groups.

Among those that received L2 therapy and were resistant to L1 platinum-basedtherapy (initiated L2 therapy ≤3 months from the cessation of L1 therapy), all patients (n = 6) were switched to a different L2 treatment and did not receive re-challenge with L1 platinum-based treatment (data not shown).

Median survival after initiation of L2 for platinum-sensitive patients was numerically longer though results were non-significant between those re-challenged with L1 platinum-based regimen (n = 20, OS = 9.1 mo (7.4–18.8)) and those switched to a different therapy (n = 13, OS = 6.4 mo (5.1-NR)), for the difference between the treatment options (Figure 2). Demographic and clinical variables were similar for platinum-sensitive patients between those re-challenged and switched (data not shown).

### 3.3. Sensitivity Analyses

As a sensitivity analysis, we analyzed all patients with sensitive disease together (*n* = 67). We found the patients re-challenged with platinum-based regimen (*n* = 27) to have a significantly longer median OS (9.1 mo, 95% CI: 6.1–12.1) than those switched to topotecan (*n* = 32, 4.6 mo, 95% CI: 2.9–6.2, *p* < 0.001). Four patients received PD-1/PD-L1 inhibitor therapy and had a median OS of 16.4 mo (95% CI: NR).

Additionally, a multivariable model considering of all L2 patients (focusing on OS from initiation of L2 therapy) incorporated age, sex, stage (at initial diagnosis), initial PS, brain metastasis, platinum sensitivity to L1 and treatment regimen at L2, found that extensive stage patients had a HR for death of 2.05 (95% CI: 1.19–3.53) as compared to limited stage, and patients that received treatment with topotecan had a HR of 2.67 (95% CI: 1.47–4.85) as compared to those re-treated with platinum based regimen (data not shown).

A further sensitivity analysis examined OS from initiation of L2 therapy stratified by the relapse free interval from end of L1 therapy to initiation of L2 therapy (split by 0–3 months, 3–6 months, 6–9 months and 9–12 months). No difference in OS was observed between the different relapse free intervals.

We further focused on 39 patients (16.6% of the study cohort) who survived for at least 24 months from initiation of L1 treatment. Of these, 80% were limited stage, 54% were female and 72% had 0–1 PS. Of the 8 patients with initial extensive stage disease who demonstrated long-term survival, 5 (63%) were female, 7 (88%) had 0–1 PS, 6 (75%) received radiation during L1 treatment (who had excellent response of mostly advanced localized disease), and 7 (88%) were brain metastases free.

## 4. Discussion

Our study reports patient characteristics and OS for a consecutive cohort of unselected SCLC patients. To the best of our knowledge, this is the first real-world study of SCLC patients with relatively recent data, including some patients that received immunotherapy as L2 therapy. In our study, only 43% of patients received L2 therapy and even fewer (12%) went on to receive L3 therapy.

Median OS from L1 treatment initiation was 11.8 months, similar to previous studies [51,52,53,54], a result that has not significantly changed over the past 20 years. OS for extensive stage patients was 9.1 months (95% CI: 8.4, 10.3), comparable to a recent study which reported 10.7 months (95% CI: 9.3, 11.8) [44] in a real-world setting, as well as the control arm of a clinical study which reported 10.3 mo (95% CI: 9.3, 11.3) [12]

Predictors of survival included better PS for extensive stage patients, and female sex for limited stage patients, and were mostly similar to those reported in previous studies [55,56,57,58]. Unlike studies which identified female sex as positive prognostic factors for all patients, in our cohort the benefit was seen only for the limited disease patients who dominated the long-term survivors. Other prognostic factors reported in the literature include PS at the time of disease recurrence for all patients [59] and sensitivity to L1 therapy [31,60].

Median OS for patients from L2 treatment was 4.5 months for extensive stage and 8.7 months for limited stage, similar to other studies [31,61] showing the important prognostic significance of stage at initial diagnosis. We found when limiting the analysis to platinum-sensitive patients, those re-treated with platinum–based chemotherapy had a longer numerical survival than those switched to treatment with topotecan single agent for both extensive and limited stage, but did not reach statistical significance, similar to a previously published study [31]. A randomized phase III prospective trial including 180 platinum sensitive patients were treated at relapse with platinum-based regimen or other chemotherapy. Those re-treated with platinum-based therapy had better outcomes than those that were switched [62]. The non-statistical significance in our study could be due to the small numbers in the sub-cohorts. L2 therapy is generally much less effective than initial treatment making it more challenging to draw conclusions on optimal treatment.

Finally, we compared OS from initiation of L2 treatment, by relapse free interval from end of L1 treatment to initiation of L2 treatment, however did not find any difference in OS between the groups, similar to another study [63].

OS for SCLC has not improved over the last few decades, and in recent years immunotherapy has become a potential new treatment for SCLC patients. In 2019 the US Food and Drug Administration approved atezolizumab in combination with L1 platinum doublet chemotherapy for extensive stage disease, and recently durvalumab was approved in a similar setting, based on improvement in OS and progression free survival [12,64]. Although the numbers were small, we found that those switched to immunotherapy had a longer numeric median OS than those that received platinum-basedor topotecan therapy for extensive stage. These results need to be interpreted with caution but may be seen as supportive for the role of immunotherapy in SCLC treatment.

Long-term survivors were more likely to be female, have limited stage and 0–1 PS, at baseline and [31] received up-front radiotherapy similar to reports in the literature [65,66].

This study has several strengths including high quality data obtained from the MHS digital database and comprehensive review of patient medical records, long follow up and relatively recent data. Limitations include the retrospective nature of this study with a lack of data as to treatment decisions and patient treatment preference in L2.

## 5. Conclusions

OS for SCLC patients in a real-world setting was found to be similar to those reported in clinical trials. Factors significantly associated with prognosis included PS for extensive stage patients and sex for limited stage patients. Re-challenge of the platinum-based doublet in relapsing patients who were platinum sensitive was associated with longer OS compared to switching to topotecan treatment in extensive stage patients. In this cohort, long term (24 months or more) survivors, were associated mostly with female limited stage patients, with low PS and upfront combined chemotherapy-irradiation approach.

## Figures and Tables

**Table 1:** Demographic and clinical characteristics of small cell lung cancer (SCLC) patients at index date (initiation of L1 treatment), n = 235. n(%) unless otherwise stated.

		Total *n* = 235	Extensive Stage *n* = 150	Limited Stage *n* = 85	*p*-Value
Demographic Variables					
Sex	Female	91 (38.7)	57 (38.0)	34 (40.0)	0.870
Age	median (IQR **)	64.0 (58.0,69.5)	64.0 (58.0,69.0)	63.0 (58.0,70.0)	0.637
	35–64	124 (52.8)	76 (50.7)	48 (56.5)	0.684
	65–74	86 (36.6)	57 (38.0)	29 (34.1)	
	≥75	25 (10.6)	17 (11.3)	8 (9.4)	
District	Centre	155 (66.0)	104 (69.3)	51 (60.0)	0.245
	North	50 (21.3)	27 (18.0)	23 (27.1)	
	South	30 (12.8)	19 (12.7)	11 (12.9)	
Socio-economic status, groups *	Low	101 (43.0)	65 (43.3)	36 (42.4)	0.933
	Median	49 (20.9)	32 (21.3)	17 (20.0)	
	High	85 (36.2)	53 (35.3)	32 (37.6)	
Clinical Variables					
Co-morbidities	Diabetes mellitus	70 (29.8)	45 (30.0)	25 (29.4)	1.000
	Cardiovascular disease	72 (30.6)	48 (32.0)	24 (28.2)	0.650
	Hypertension	132 (56.2)	83 (55.3)	49 (57.6)	0.836
	Chronic kidney disease	40 (17.0)	27 (18.0)	13 (15.3)	0.727
	COPD	75 (31.9)	50 (33.3)	25 (29.4)	0.635
	Osteoporosis	43 (18.3)	29 (19.3)	14 (16.5)	0.712
Smoking	Ever	224 (95.3)	141 (94.0)	83 (97.6)	0.342
	Never	11 (4.7)	9 (6.0)	2 (2.4)	
ECOG PS	0–1	141 (60.0)	85 (56.7)	56 (65.9)	0.039
	2	30 (12.8)	25 (16.7)	5 (5.9)	
	3–4	4 (1.7)	4 (2.7)	0 (0.0)	
	Unknown	60 (25.5)	36 (24.0)	24 (28.2)	
Brain metastases		26 (11.1)	26 (17.3)	0 (0.0)	<0.001

* Socioeconomic status is based on the poverty index of the member’s enumeration area, as defined by the 2008 National Census. The poverty index is based on parameters including household income, educational level, crowding, physical conditions and car ownership. A higher score indicates higher level of socioeconomic status. ECOG, eastern cooperative oncology group; PS, performance status; COPD chronic obstructive pulmonary disease. ** IQR = interquartile range

**Table 2:** Univariate analysis for prognostic parameters for overall survival (survival from initiation of L1 treatment, unless otherwise stated; log-rank test).

Prognostic Parameters		N	Events n (%)	Median OS (95% CI)	Alive At 1 Year N (%)	Alive at 2 Years N (%)	*p*-Value
EXTENSIVE STAGE, N = 150							
Sex	Male	93	84 (90.3%)	8.91 (7.96,10.50)	30.0% (21.8–41.4%)	3.9% (1.3–11.8%)	0.13
	Female	57	51 (89.5%)	9.63 (8.61,11.70)	32.8% (22.6–47.8%)	13.6% (6.7–27.7%)	
ECOG PS	0–1	85	73 (85.9%)	9.63 (8.65,13.80)	41.0% (31.6–53.2%)	11.2% (5.7–22.0%)	0.09
	2	25	25 (100.0%)	8.91 (4.9,11.30)	20.0% (9.1–43.8%)	-	
	3–4	4	4 (100.0%)	8.43 (3.45, NR)	-	-	
	Unknown	36	33 (91.7%)	8.73 (6.94,9.90)	19.7% (9.9–39.2%)	6.6% (1.7–24.7%)	
Brain metastases	No	124	111 (89.5%)	9.17 (8.38,10.30)	32.1% (24.7–41.8%)	7.6% (3.8–15.2%)	0.89
	Yes	26	24 (92.3%)	8.81 (7.27,12.10)	26.9% (14.3–50.7%)	7.7% (2.0–29.1%)	
Survival from line	L1	150	135 (90.0%)	9.14 (8.38, 10.30)	31.08% (24.3–39.7%)	7.70% (4.2–14.2%)	
	L2	62	57 (91.9%)	4.54 (3.19, 6.12)	9.99% (4.4–22.5%)		
Treatment pattern at L2 for those sensitive to L1 treatment	Retreatment	7	7 (100.0%)	6.81 (6.12, NR)	14.30% (2.3–87.7%)		0.06
	Treatment switch	27	23 (85.2%)	4.5 (2.63, 6.61)	7.06% (1.3%–39.7%)		
**LIMITED STAGE, N = 85**							
Sex	Male	51	39 (76.5%)	21.50 (17.60,27.10)	79.9% (69.4–91.9%)	38.0% (26.1%55.3%)	0.03
	Female	34	16 (47.1%)	29.60 (20.4, NR)	75.8% (62.5–92.0%)	57.9% (42.5–78.7%)	
ECOG PS	0–1	56	33 (58.9%)	24.50 (20.50,34.90)	85.0% (75.9–95.2%)	50.5% (38.0–67.2%)	0.07
	2	5	3 (60.0%)	50.50 (22.10, NR)	80.0% (51.6–100.0%)	60.0% (29.3–100.0%)	
	Unknown	24	19 (79.2%)	16.00 (11.70,32.30)	62.5% (45.8–85.2%)	31.4% (17.0–58.1%)	
Survival from line	L1	85	55 (64.7%)	23.54 (19.40,27.90)	78.20% (69.8–87.7%)	45.70% (35.6–58.5%)	
	L2	39	33 (84.6%)	8.68 (6.02, 14.04)	34.37% (21.9–54.0%)	7.50% (2.1–27.1%)	
Treatment pattern at L2 for those sensitive to L1 treatment	Retreatment	20	17 (85.0%)	9.11 (7.40, 18.80)	36.94% (20.5–66.5%)	12.31% (3.4–44.1%)	0.14
	Treatment switch	13	12 (92.3%)	6.43 (5.06, NA)	25.00% (9.4–66.6%)		

ECOG, eastern cooperative oncology group; PS, performance status;NR, not reported

**Table 3:** Multivariable model for factors associated with survival from L1 treatment initiation for the study cohort (N = 235).

		Adjusted HR	95% CI		*p-*Value
			Lower	Upper	
EXTENSIVE STAGE, N = 150					
Age	years	1.01	0.99	1.04	0.191
Sex	Male vs. female	1.32	0.92	1.92	0.136
Socio-economic status, groups *	low (Ref.)				
	Medium	0.77	0.47	1.25	0.283
	High	0.78	0.53	1.16	0.225
ECOG PS	0–1 (Ref.)				
	2	1.63	1.00	2.65	0.048
	3–4	2.45	0.83	7.24	0.104
	Unknown	1.53	0.99	2.35	0.054
Brain metastases		0.98	0.62	1.54	0.927
**LIMITED STAGE, *n* = 85**					
Age	years	0.99	0.95	1.02	0.474
Sex	Male vs. female	2.17	1.12	4.20	0.022
Socio-economic status, groups *	low (Ref.)				
	Medium	0.93	0.46	1.87	0.845
	High	0.71	0.36	1.42	0.337
ECOG PS	0–1 (Ref.)				
	2	0.75	0.21	2.63	0.650
	Unknown	2.42	1.31	4.47	0.005

* Socioeconomic status is based on the poverty index of the member’s enumeration area, as defined by the 2008 National Census. The poverty index is based on parameters including household income, educational level, crowding, physical conditions and car ownership. A higher score indicates higher level of Socioeconomic status. ECOG, eastern cooperative oncology group; COPD chronic obstructive pulmonary disease.

**Table 4:** Multivariable model for factors associated with survival from L2 treatment initiation (n = 101).

		Adjusted HR	95% CI		*p-*Value
			Lower	Upper	
EXTENSIVE STAGE, N = 62					
Age *	years	1.01	0.98	1.05	0.531
Sex	Female vs. Male	1.55	0.83	2.91	0.172
ECOG PS *	0–1 (Ref.)				
	2	2.22	1.03	4.79	0.043
	3–4	3.45	0.36	33.16	0.284
	missing	1.33	0.62	2.85	0.462
Brain metastases *		0.76	0.36	1.60	0.471
L2 Drug Treatment	Platinum-based regimen (Ref.)				
	Topotecan	2.79	1.05	7.39	0.039
	PD-1/PD-L1 inhibitor therapy	0.96	0.27	3.40	0.950
	Other	1.27	0.35	4.66	0.715
Platinum sensitivity to L1 treatment		1.18	0.64	2.2	0.592
**LIMITED STAGE, n = 39**					
Age *	years	1.01	0.95	1.07	0.762
Sex	Female vs. Male	1.08	0.41	2.84	0.870
ECOG PS *	0–1 (Ref.)				
	2	0.3	0.03	2.63	0.274
	missing	2.28	0.89	5.86	0.087
L2 Drug Treatment	Platinum-based regimen (Ref.)				
	Topotecan	2.43	0.94	6.24	0.066
	PD-1/PD-L1 inhibitor therapy	0.19	0.02	2.24	0.186
	Other	0.23	0.04	1.17	0.076
Platinum sensitivity to L1 treatment		0.33	0.08	1.40	0.134

* At diagnosis. ECOG, eastern cooperative oncology group; PS, performance status.
